# Reliability of an Innovative Slab Shear versus Microtensile Bond Strength Test: Mechanical and Finite Element Analysis

**DOI:** 10.1055/s-0043-1763498

**Published:** 2023-04-14

**Authors:** Emad Abd Elfatah Abo-Alazm, Ahmed Abdou, Layla Hassouneh, Rehab Khalil Safy

**Affiliations:** 1 Department of Restorative Dentistry, Egyptian Russian University, Badr City, Cairo Governorate, Egypt; 2 Department of Prosthetic Dentistry, Biomaterials Division, Faculty of Dentistry, King Salman International University, El Tur, South Sinai, Egypt; 3 Department of Conservative Dentistry, Jordan University of Science and Technology, Ar-Ramtha, Jordan; 4 Department of Conservative Dentistry, Suez Canal University, Ismailia Governorate, Egypt

**Keywords:** slab shear, microtensile, pretest failure, bond strength test, failure mode, finite element analysis

## Abstract

**Objective** The aim of this study was to evaluate the efficiency of slab shear bond strength test (Slab_SBS) versus the microtensile in evaluation of the bond strength of different substrates.

**Materials and Methods** Forty-eight extracted caries-free human third molars were utilized for teeth specimens' preparation. After flattening of all molars' occlusal table, the specimens were divided into two groups based on the type of utilized restorative material: nanohybrid resin composite and resin-modified glass ionomer (RMGI). Each group was further subdivided into three subgroups according to the subsequently applied bond strength test and specimen width; microtensile bond strength test (μTBS), Slab_SBS [2 mm] and Slab_SBS [3 mm]. Both testing methods were additionally applied on CAD/CAM specimens, nanohybrid resin composite blocks (composite-to-composite), and ceramic blocks (ceramic-to-ceramic). CAD/CAM specimens were prepared and cemented and then sectioned and subdivided as followed for teeth specimens' preparation. Pretest failures (PTF), bond strength, and failure mode of each specimen were recorded. Representative three-dimensional (3D) finite element analysis (FEA) models were developed to simulate μTBS and Slab_SBS specimens. Data were statistically analyzed using Shapiro–Wilk test and Weibull analysis.

**Results** Pretest failures were only noted in the μTBS subgroups. Slab_SBS provided comparable bond strength to the μTBS of all substrates with adhesive mode of failure.

**Conclusion** Slab_SBS is easier to prepare with consistent and predictable outcome with no pretest failures during specimen preparation and better stress distribution.

## Introduction

The trend toward a more image-focused society has resulted in a significant increase in the demand for aesthetic restorations. Efficient adhesives that form durable bonds between the dental substrate and the bonded restoration are frequently essential to the longevity and effectiveness of contemporary dental restorations. Reliable laboratory methods are mandatory to forecast the clinical outcomes of such restorative treatment. Such tests offer a general pattern of prediction on how bonded restorations could perform in clinical scenarios. Nevertheless, 'The more realistic' clinical performance reported by laboratory tests still does not reflect the actual clinical outcomes. 1 2 3 Therefore, concerns that in vitro bond strength assessments are insufficient predictors of clinical success 4 5 6 7 in conjunction with different results from tests utilizing various equipment for bond strength studies, 8 9 necessitate the ongoing search for innovative, more uniform and reproducible testing method to obtain comparable data.

Microtensile bond strength test (μTBS) is one of the most commonly used methods. Since its invention by Sano et al (1994), it has been frequently utilized to assess the efficiency of bonding to various dentin substrates 10 11 12 13 14 with/and other various substrates including ceramics 15 16 17 , resin composite, 18 19 20 21 22 23 24 and glass ionomer 25 26 . Despite the fact that μTBS is currently regarded as a standardized, reliable, and versatile test to evaluate bond strength regardless of the tested material, 11 27 the method's benefits are somewhat outweighed by its labor-intensive nature, requirement for high technical proficiency, and rapid dehydration of small sized specimens throughout handling. 28 In addition, it is crucial to note that μTBS resulted in some warranted criticism in recording pretesting failure, which is still occurring as a significant problem for such testing method with its subsequent implications. 29 Nevertheless, μTBS has been criticized for being a labor-intensive procedure; it enables the preparation of several specimens from each tooth. 30 There is a trade-off between the more labor required to use this method and the additional data that can be acquired from each tooth. Consequently, searching for an innovative laboratory screening test method that is versatile, reliable, standardized, less labor intensive, and provides nearly the same number of specimens could be a breakthrough in bond strength evaluation.

As understanding the force distribution and stress patterns, which eventually impact the mode of failure, is essential when assessing the efficacy of a specific bond strength test, 31 finite element analysis (FEA) has recently been recommended to gain foothold in bond strength studies, through evaluation of stress distribution patterns.

Therefore, the purpose of this study was to evaluate the reliability of an innovative testing method termed slab shear bond strength test (Slab_SBS) in measuring the bond strength of different substrates in comparison to the μTBS approach and validating the bond strength values obtained using both tested methods by the FEA. The null hypotheses tested of the current study were: (1) the slab_SBS and the microtensile bond strength results would not differ among each other when used for evaluation of bond strength of different substrates and (2) both bond strength testing methods would have the same mode of failure.

## Materials and Methods

All tested materials and their description, composition, manufacturers, and batch number are displayed in Table 1 .

**Table 1:** Materials, description, composition, manufacturers, and batch numbers

Material	Description	Composition	Manufacturer	batch numbers
Tetric N-bond Universal	Universal adhesive	Methacrylates, ethanol, water, highly dispersed silicon dioxide, initiators, and stabilizers.	Ivoclar Vivadent, Schaan, Liechtenstein.	Z01WS9
Tetric N-Ceram, shade A2	Nanohybrid resin composite.	Matrix: Bis-GMA, UDMA, TEGDMA, Bis-EMA resins Filler: Barium glass, ytterbium trifluoride, mixed oxide, silicon dioxide, Prepolymers, Nanofillers. Filler loading 81 (wt%), 57 (vol%)	Ivoclar Vivadent, Schaan, Liechtenstein.	Z01 × 78
Dentin Conditioner	Mild poly acrylic solution	10% poly acrylic, 90% distilled water	GC, Tokyo, Japan.	1601151
Fuji II LC	Light-cured Resin modified Glass Ionomer Restorative, shade A2	.powder: fluoroaluminate silicate glass particles. Liquid: copolymers of polyacrylic acid and maleic acid, HEMA, water, camphorquinone, photoinitiator,	GC, Tokyo, Japan.	2110252
Fuji coat LC	Light cured low viscosity resin	Methylmethacrylate, multifunctional methylmethacrylate camphorquinone, photoinitiator.	GC, Tokyo, Japan.	2003165
Grandio Blocs	Nanohybrid resin composite CAD/CAM blocks, shade A2	Resin: Bis-GMA, TEGDMA. Filler: Ba–Al–Si glass/Silica nanoparticles 89% by weight and 71.4%by volume with a particle size range of 20–40 nm	VOCO GmbH Cuxhaven, Germany	1950657
IPS e.max CAD, shade A2	lithium disilicate glass-ceramic CAD/CAM blocks.	>57% SiO _2_ , Li _2_ O, K _2_ O, P _2_ O _5_ , ZrO _2_ , ZnO, Al _2_ O _3_ , MgO, pigments	Ivoclar Vivadent, Schaan, Liechtenstein.	R51558
IPS ceramic etching gel	ceramic etching gel	5% hydrofluoric acid, water, thickener, surfactant and dye	Ivoclar Vivadent, Schaan, Liechtenstein.	V37045
Panavia SA cement universal	self-adhesive resin cement	Paste A: Monomer (10-MDP, Bis-GMA, TEGDMA, HEMA, other methacrylate monomer), filler (silanated barium glass filler, silanated colloidal silica), initiator, pigment, others Paste B: Methacrylate monomer, filler (silanated barium glass filler, aluminum oxide, silanated sodium fluoride), accelerator, pigment, silane coupling agent, others	Kuraray Noritake Dental	A80067

Abbreviations: Bis-EMA, ethoxylated bisphenol-A dimethacrylate; Bis-GMA, bis-phenol A diglycidyl methacrylate; CQ, camphorquinone; HEMA, hydroxyl ethyl methacrylate; TEGDMA, tri ethylene glycol dimethacrylate; UDMA, urethane dimethacrylate.

**1. Preparation of [composite-to-tooth] and [RMGI-to-tooth] groups**
**1. A. Teeth specimens' Preparation**

**Fig. 1:**
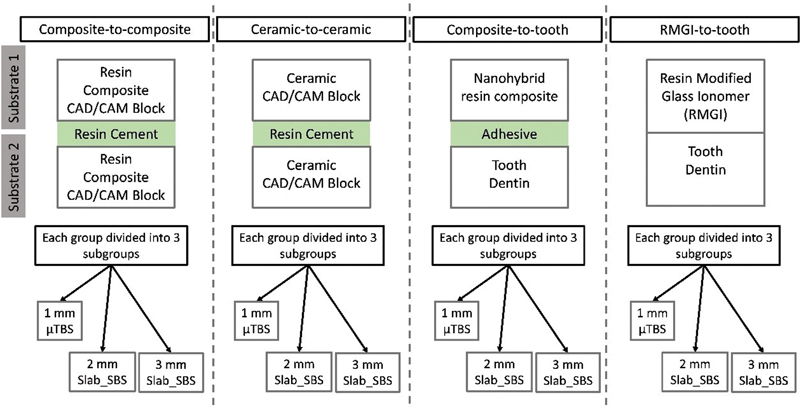
Flowchart of experimental groups.

Forty-eight human impacted, crack-free third molars from patients between the ages of 20 and 30 years were used for the current study. Teeth were collected following informed consent granted by the Research Ethics Committee (REC), Faculty of Dentistry, Suez Canal University (ethical approval No. 542/2022). Following the removal of any soft tissue remnants, teeth were kept in distilled water containing 0.2% Thymol for no more than 3 months at 4°C before testing. Each tooth was fixed in an acrylic resin block, after which the occlusal surface of each molar was flattened 1 mm behind the DEJ using an automated diamond saw (Isomet 4000, Buehler Ltd., Lake Bluff, United States). The specimens were then divided into two groups ( *n* = 24) based on the type of restorative material that was used; nanohybrid resin composite (Tetric N-Ceram, Ivoclar Vivadent, Schaan, Liechtenstein) (composite-to-tooth) group, and resin-modified glass ionomer (Fuji II LC, GC, Tokyo, Japan) (RMGI-to-Tooth) group ( Fig. 1 ).

Building up of both restorative materials was performed through utilization of two especially constructed Teflon molds. Molds dimensions were 8 mm length, 3 mm thickness, and two variable widths (2 mm) and (3 mm). Thus, each group was further subdivided into three subgroups ( *n* = 8), according to the subsequently applied bond strength test and specimen width; μTBS (1 mm), Slab_SBS (2 mm) and Slab_SBS (3 mm) width, respectively. Restoration of the composite-to-tooth group was preformed through the application of universal adhesive (Tetric N-bond Universal, Ivoclar Vivadent, Schaan, Liechtenstein) that was applied to the dentin surface and agitated for 20 seconds, air-blown for 5 seconds according to the manufacturer's instructions. The adhesive was then light cured for 10 seconds using an LED-curing device with a light intensity of 1600 mW/cm ^2^ and a wavelength range of 450 to 480 nm (Ortholux Luminous, 3M, ESPE). The 14 mm-diameter LED guide tip was kept at zero distance from the specimen surface throughout curing. The LED unit's built-in radiometer was used to periodically verify the light intensity. Following the bonding procedure, Teflon mold was positioned centrally on the bonded dentin surface, and incremental buildup of the resin composite was conducted using two layers of the nanohybrid resin composite, each with a thickness of 1.5 mm. This buildup was then incrementally light-cured for 40 seconds in accordance with the manufacturer's instructions.

On the other side, dentin surfaces conditioning was carried out for RMGI-to-tooth group specimens. In accordance with the manufacturer's instructions, dentin surfaces received a 10 second application of cavity conditioner (Cavity Conditioner, GC, Tokyo, Japan) before being rinsed and gently dried. Afterward, RMGI capsules were activated, mechanically mixed for 7 seconds according to the manufacturer's instructions, injected inside the centrally seated Teflon molds and light cured for 20 seconds. Once set, the mold was removed, and a final coat (Fuji coat, GC, Tokyo, Japan) was painted to the surface and light cured for 10 seconds. Then, all bonded specimens were stored in distilled water for 24 hours at room temperature before testing procedures.

**1. B. Microtensile bond strength testing**

After the storage time, a diamond saw was utilized to serially section each μTBS specimen (8 × 3 × 3 mm) in *“x” and “y”* directions into rods (1 mm) thick, (1 mm) wide, and (3 mm) long), and defective rods were recorded as PTF and excluded. All rods' dimensions were verified using a digital caliper (Absolute Digimatic, Mitutoyo, Tokyo, Japan) with a 100 μm accuracy. 14 To avoid the adverse consequences of excess or lack of adhesive at the interface on results, five middle rods were selected to evaluate the μTBS of each specimen with a total of 40 rods for each subgroup. A cyanoacrylate adhesive was utilized to attach each rod to the attachment jig from either ends to ensure that the bonded interface would be exactly in the middle of the two proximal ends of the jig. After that, it was tested in a universal testing machine (Instron, model 3345, England) at a crosshead speed of 1.0 mm/minute until rod failure. Results in units of mega Pascal (MPa), were calculated and recorded by a computer software (BlueHil universal Instron, England) as the maximum tensile load (Newton) was divided by a cross-sectional area of the rod of about 1.0 mm ^2^ .

**1. C. Slab shear bond strength test**

All specimens of both Slab_SBS subgroups (8 × 3 × 2 mm) and (8 × 3 × 3 mm) were sectioned in one direction (width direction) using a diamond saw to produce bonded slabs (1 mm] thick, (3 mm) long, of either (2 mm) or (3 mm) width. Each specimen yielded five slabs, totaling 40 slabs for each subgroup ( *n* = 40 slabs/subgroup) and the PTF was recorded. Each tooth part of each slab was mounted to a special attachment, at a distance of 0.5 mm from the bonded interface, fixed to the lower head of testing machine. Compression mode of force was delivered using a shearing blade (Chisel) attached to the upper movable testing head at a crosshead speed of 1 mm/minute until specimen failure. The shearing force was applied as close as possible to the bonded interface. Shear bond strength in MPa was calculated by dividing the load over the respective cross-sectional area (2 mm ^2^ ) or (3 mm ^2^ ) of each slab. Steps for slab-SBS specimen preparation can be checked in [ Supplementary Fig. S1 , available in the online version].

**
2. Preparation of [composite-to-composite] and [ceramic-to-ceramic] groups ( Figure 1 )
**
**2. A. CAD/CAM Specimen Preparation**

Two main groups of CAD/CAM blocks measuring 12 mm × 14 mm × 18 mm were involved ( *n* = 48); composite-to-composite group of nanohybrid resin composite (Grandio Blocs, VOCO GmbH Cuxhaven, Germany) and ceramic-to-ceramic group (IPS e.max, Ivoclar Vivadent, Schaan, Liechtenstein). All blocks were longitudinally sectioned in the x and y-axes, followed by horizontal sectioning with a low-speed diamond saw under copious water irrigation. Small blocks measuring (10 mm] length, (5 mm) width, and (4 mm) thickness were retrieved to be used as the base and top blocks measuring (8 mm) length, (3 mm) thickness, with two variable widths (2 mm) and (3 mm). The bonded surfaces of all sectioned CAD/CAM blocks were manually wet ground using #600 grit SiC paper for 10 seconds to standardize the roughness of specimens. 19

The resin composite CAD/CAM blocks bonded surfaces were air-abraded with 50 µm Al _2_ O _3_ (MicroBlaster; bio-art, Sao Carlos, Brazil) at 0.2 MPa pressure with an angle of 45 degrees at 10 mm distance for 10 seconds. According to the manufacturer's recommendations, each air-abraded resin composite surface was cleaned with sterile cotton (Cotton Buds, Cotton Stick, Egypt), soaked in 70% medicinal alcohol, and air dried with oil/water free compressed air for 10 seconds.

Preparation of the bonded surfaces of the ceramic CAD/CAM blocks were performed through conditioning by hydrofluoric acid (Ivoclar Vivadent AG, Schaan, Lichtenstein) for its superior retention performance, as shown in multiple studies. 32 33 34 5% hydrofluoric acid was applied for 20 seconds and then rinsed for 20 seconds. 35 After that, all specimens underwent a 5-minute ultrasonic cleaning and gently air-dried for 5 seconds.

**2. B. Cementation of CAD/CAM blocks**

The mixed self-adhesive resin cement (Panavia SA cement Universal, Kuraray Noritake Dental) was injected on the prepared surface of each CAD/CAM base block without silanization according to its manufacturer's instructions, followed by positioning of either resin composite or ceramic top block according to each group. Each cemented block was positioned in the loading device for 2 minutes using 500 gm static load. 34 Using a disposable micro brush, excess resin cement was carefully removed from each side of the cemented blocks during loading period. Each specimen was unloaded then light cured for 40 seconds at right angles to each margin. Thereafter, according to the aforementioned top block widths and the subsequent bond strength test, each group was further subdivided into three subgroups ( *n* = 8) as previously mentioned. Finally, all specimens were stored in distilled water for 24 hours at room temperature before testing procedures.

**2. C. Microtensile bond strength testing**

Following the storage period, each specimen of μTBS subgroups was sectioned into rods ( *n* = 40), PTF was recoded and excluded, and then its bond strength was measured as mentioned before.

**2.D. Slab shear bond strength test**

**Fig. 2:**
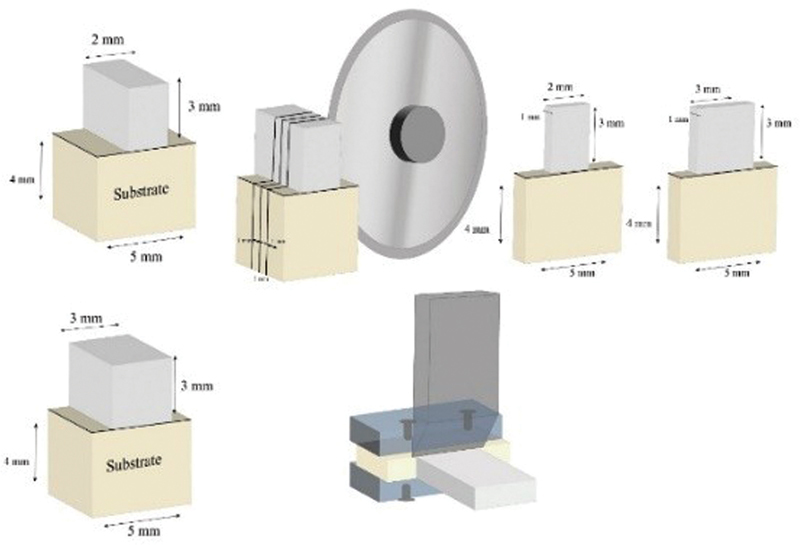
Schematic diagram for the sectioning process of slab shear bond strength test For 2 mm and 3 mm width specimens.

**Fig. 3:**
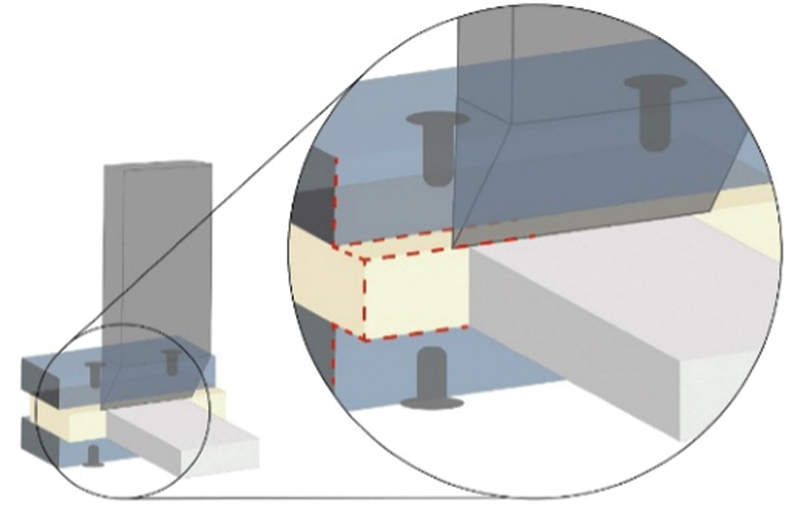
Schematic diagram for the loading of the slab shear bond strength test.

**Fig. 4 A and B:**
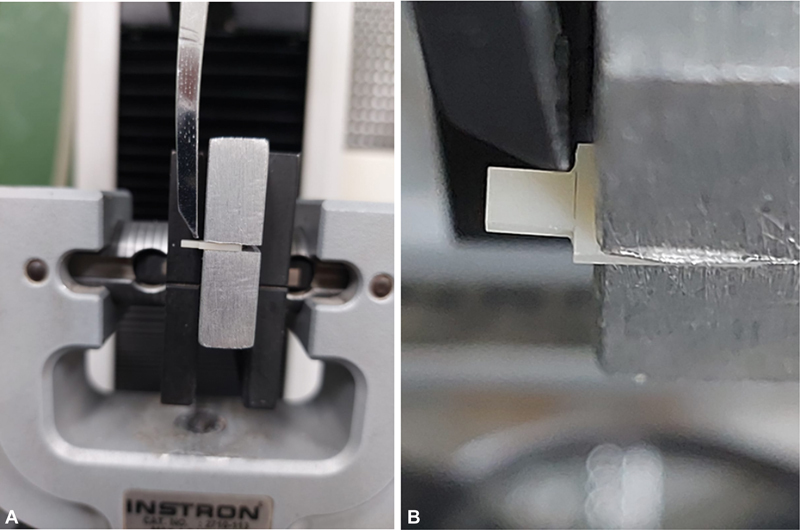
Application of shear stress as close as possible to the interface of the slab shear bond strength test slab.

After storage, all specimens of all Slab_SBS subgroups (2 mm) and (3 mm) width were sectioned across its width direction using the Isomet disc into (1 mm) thick slabs ( Fig. 2 ). After recording the PTF if present, five slabs were retrieved from each specimen ( *n* = 40 slabs/subgroup). Slab_SBS of each slab was measured as mentioned before **(** Figs. 3 , 4A and 4B ).

**3. Failure mode analysis**

Failure mode of each rod or slab was examined using a stereomicroscope (25 × ; Olympus, Tokyo, Japan) and classified as the following 19 36 : adhesive (A): failure at bonding interface, cohesive (C): failure within dentin or restorative material, and mixed (M): failure at bonding interface with fragments of dentin or restorative material.

### Statistical Analysis

Bond strength showed a parametric distribution when checked using the Shapiro–Wilk test. Pretest failures (ptf) were treated as left censored data. Bond strength data were analyzed using the Weibull analysis (R4, R Foundation for statistical computing, Vienna, Austria). Weibull parameters were calculated using the maximum likelihood estimation, and 95% confidence intervals were calculated with Monte Carlo simulations. The different groups were compared at the characteristic strength (63.2% and 10% probability of failure) in addition for Weibull modulus. A significant level was set at 0.05 (α = 0.05).

## Finite Element Analysis

**Fig. 5:**
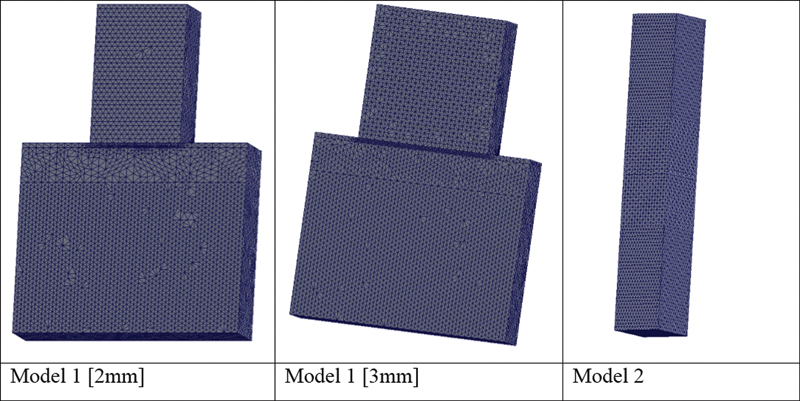
Representative image of the mesh design of models 1 (2 mm and 3 mm) and models 2.

Twelve representative FEA models were created (SolidWorks 2020, Dassault Systèmes, SolidWorks Corps). Each model's geometry and structure were designed to simulate the dimensions of the tested specimens. The Slab_SBS specimens were designed as model 1 (2 mm) and model 1 (3 mm) and μTBS specimen was designed as model 2 ( Fig. 5 ). All experimental models' meshing was created using parabolic tetrahedral solid elements. The meshes were constructed using a 5% strain energy and displacement variation convergence test. The number of total nodes and elements in experimental models were 253,026 nodes and 175,635 elements for model 1 (2 mm), 314,852 nodes, 219,105 elements for model 1 (3 mm); and 205,198 nodes, 141,919 elements for model 2. All restorative materials used in the current investigation and the dentin structure were considered to be linear, elastic, homogeneous, and isotropic. According to the literature 37 38 39 40 41 42 43 44 45 , the mechanical parameters of the tested materials are shown in Table 2 .

**Table 2:** Mechanical properties adopted for the simulated tested materials

Tooth structure/materials	Young's modulus (GPa) *	Poisson's ratio	Flexural strength (Mpa)	Micro hardness	Density (g/cm ^3^ )
Dentin	18.6	0.31	212.9 ± 41.9	66.3 ± 5.7	1.7 ± 0.02-2.12 ± 0.03
Grandio Blocs	18	0.26	271 ± 26	130.6	2.2
Panavia SA cement universal	17	0.3	N/A	N/A	N/A
IPS e.max CAD	102.7	0.22	530	545.68	2.5 ± 0.1
Tetric ^®^ N-Ceram	16.4	0.28	119.20 ± 20.32	45.2	2.1
Fuji II LC	10.8	0.3	104.77 ± 3.97	46.2 ± 3.18	N/A

* GPa: giga pascal.

Mpa: mega pascal.

g/cm: gram/centimeter.

N/A: not available.

The contact interfaces between all parts of the model were considered to be entirely bonded, and the cement layer's thickness was assumed to be 100 µm. Due to numerical considerations, the thin adhesive layer (10 µm) in between tooth substrate and either resin composite or RMGI could not be simulated or modeled for the FEA. 46 47 Therefore, during model creation and FEA, the thin adhesive layer was neglected in models mimicking dentin bonded to resin composite or RMGI.

Each model's boundary conditions were simulated based on the circumstances used for *in vitro* mechanical testing. To create the 3 mm loading span for model 1, a total force of 30 N was applied perpendicular to the specimen's long axis at the upper substrate surface at the bonded interface. For model 2, a combined force of 30 N was applied in opposite directions at both substrate surfaces, parallel to the long axis of the specimen 1 mm from the cement interface. Using the von Mises and Sy criteria, the qualitative stress distribution analyses were recorded. 48

## Results

**Fig. 6:**
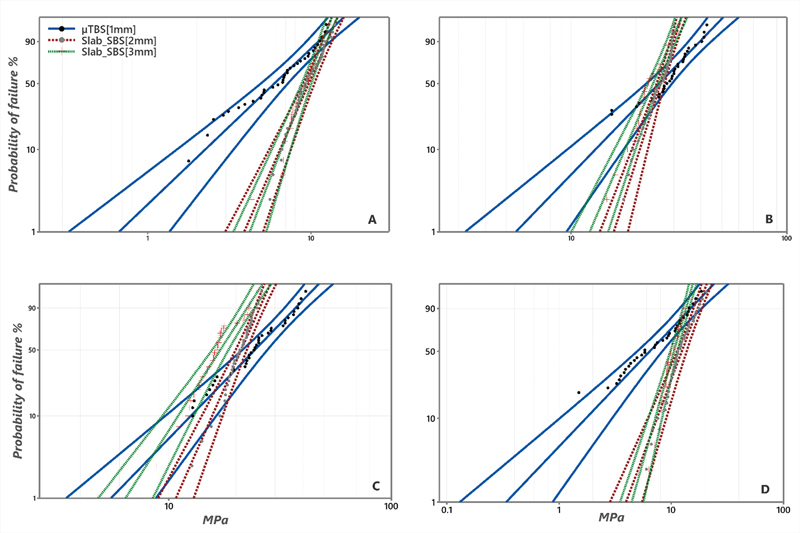
Weibull survival plot for ( **A** ) composite-to-composite substrate, ( **B** ) ceramic-to-ceramic substrate, ( **C** ) composite-to-tooth substrate, and ( **D** ) RMGI-to-tooth substrate. The shape parameter of μTBS (1 mm) showed deeper inclination compared to Slab_SBS groups for all the tested substrates in **A, B, C** , and **D** , indicating a less reliability compared to Slab_SBS.

Slab_SBS, μTBS, and failure mode analysis data are presented in Table 3 and Fig. 6 . Evaluation of the results of composite-to-composite CAD/CAM blocks revealed that μTBS (1 mm) showed the lowest significant characteristic strength and 10% probability of failure compared to both Slab_SBS subgroups. Although the modulus of Weibull parameter for Slab_SBS (2 mm) and Slab_SBS (3 mm) showed no significant difference between each other, both showed significantly higher values compared to μTBS (1 mm). Pretest failure during specimens' preparation and cutting procedures was recorded in the μTBS subgroup without any PTF in the Slab_SBS subgroups.

**Table 3:** Weibull analysis of pretest failure, bond strength, and failure mode analysis of all tested substrates

Substrate	Method/size	pft	α [95% CI]	β [95% CI]	P10 [95% CI]	FMA[A/C/M]
Composite-to-composite	µTBS [1 mm]	6/40	7.2[6.1 to 8.5] ^a^	1.9[1.5 to 2.5] ^ab^	2.1[1.5 to 3.3] ^a^	[80/5/15]
Composite-to-composite	Slab_SBS [2 mm]	0/40	10.5[9.8 to 11.3] ^bc^	4.7[3.7 to 5.9] ^cde^	6.5[5.6 to 7.5] ^b^	[100/0/0]
Composite-to-composite	Slab_SBS [3 mm]	0/40	10.1[9.5 to 10.7] ^bc^	5.3[4.2 to 6.7] ^def^	6.6[5.8 to 7.5] ^b^	[100/0/0]
Ceramic-to-ceramic	µTBS [1 mm]	10/40	29.3[26.0 to 33.0] ^h^	2.8[2.1 to 3.7] ^bc^	13.0[9.6 to 17.5] ^cd^	[100/0/0]
Ceramic-to-ceramic	Slab_SBS [2 mm]	0/40	27.3[26.1 to 28.2] ^gh^	8.5[6.6 to 10.9] ^f^	20.9[19.1 to 22.7] ^e^	[100/0/0]
Ceramic-to-ceramic	Slab_SBS [3 mm]	0/40	25.5[24.2 to 26.8] ^gh^	6.3[4.9 to 7.9] ^ef^	17.8[15.9 to 19.9] ^de^	[100/0/0]
Composite-to-Tooth	µTBS [1 mm]	4/40	27.6[24.7 to 31.1] ^gh^	2.8[2.2 to 3.7] ^bc^	12.5[9.6 to 16.3] ^cd^	[83/8/10]
Composite-to-Tooth	Slab_SBS [2 mm]	0/40	22.4[21.3 to 23.6] ^f^	6.3[5.0 to 7.8] ^f^	15.4[14.1 to 17.5] ^d^	[100/0/0]
Composite-to-Tooth	Slab_SBS [3 mm]	0/40	18.6[17.2 to 20.0] ^e^	4.3[3.4 to 5.5] ^cde^	11.0[9.4 to 12.9] ^c^	[100/0/0]
RMGI-to-Tooth	µTBS [1 mm]	7/40	8.3[6.6 to 10.4] ^ab^	1.4[1.1 to 1.9] ^a^	1.7[1.0 to 2.9] ^a^	[100/0/0]
RMGI-to-Tooth	Slab_SBS [2 mm]	0/40	13.8[12.6 to 15.1] ^d^	3.7[2.9 to 4.7] ^cd^	7.5[6.2 to 9.1] ^b^	[100/0/0]
RMGI-to-Tooth	Slab_SBS [3 mm]	0/40	11.4[10.7 to 12.2] ^c^	4.9[3.9 to 6.1] ^de^	7.2[6.3 to 8.3] ^b^	[100/0/0]

Note: Different superscript letters within (α, β and P10) columns are statistically significant based on 95% confidence interval (CI). α: characteristic strength or scale of Weibull parameter. β: the shape, slope, and modulus of Weibull parameter. P10: estimation and 95% CI at 10% probability of failure. FA; failure mode analysis (A: adhesive, C: cohesive, and M: mixed).

For ceramic-to-ceramic substrate, insignificant difference was recorded between different tested subgroups in the characteristic strength. However, the true difference was revealed in the remaining aspects of evaluation; a 25% PTF resulted for μTBS subgroup without any recorded PTF for both Slab_SBS subgroups, in addition to significant difference between μTBS (1 mm) and Slab_SBS (3 mm) at 10% probability of failure. Evaluation of the modulus of Weibull showed that Slab_SBS (2 mm and 3 mm) subgroups showed significantly higher values compared to μTBS (1 mm).

Results of the composite-to-tooth substrate demonstrated significant difference between μTBS (1 mm) and Slab_SBS [2 mm and 3 mm) in the characteristic strength. However, the μTBS subgroup showed 10% PTF, and the reliability of the bond strength was in the favor of Slab_SBS subgroups. For RMGI-to-tooth substrate, μTBS (1 mm) showed the lowest significant characteristic strength and Weibull modulus compared to Slab_SBS subgroups. Moreover, no pretest failure resulted for Slab_SBS (2 mm and 3 mm) ( Table 3 ).

**Fig. 7:**
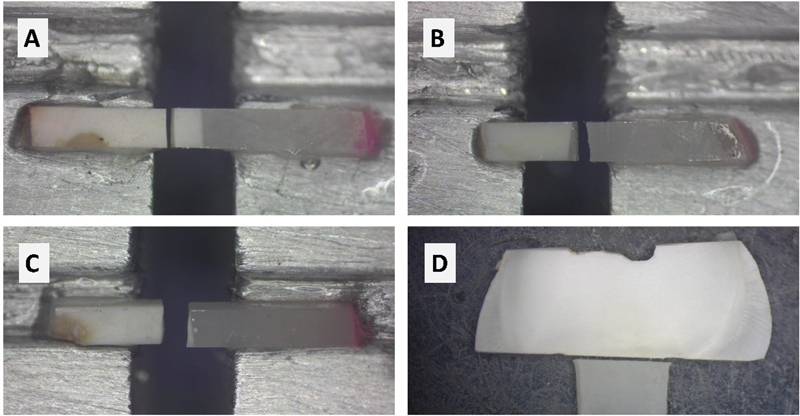
Representative images of failure mode analysis for composite-to-tooth group. ( **A** – **C** ) showing failure mode analysis for microtensile bond strength. A: Cohesive failure in dentin, B: failure at bonding interface, ( **C** ): mixed failure. ( **D** ) showing adhesive failure in Slab_SBS, which was the only failure mode resulted for Slab_SBS.

Results of failure mode analysis revealed that Slab_SBS (2 mm and 3 mm) for all substrates showed 100% adhesive failure. Meanwhile, μTBS (1 mm) showed cohesive and mixed failure for composite-to-composite substrate and composite-to-tooth. However, the ceramic-to-ceramic and RMGI to tooth groups' recorded adhesive mode of failure for all bond strength tested methods. Results of failure mode analysis and representative image of failure mode analysis is presented in Table 3 and Fig. 7 .

**Fig. 8:**
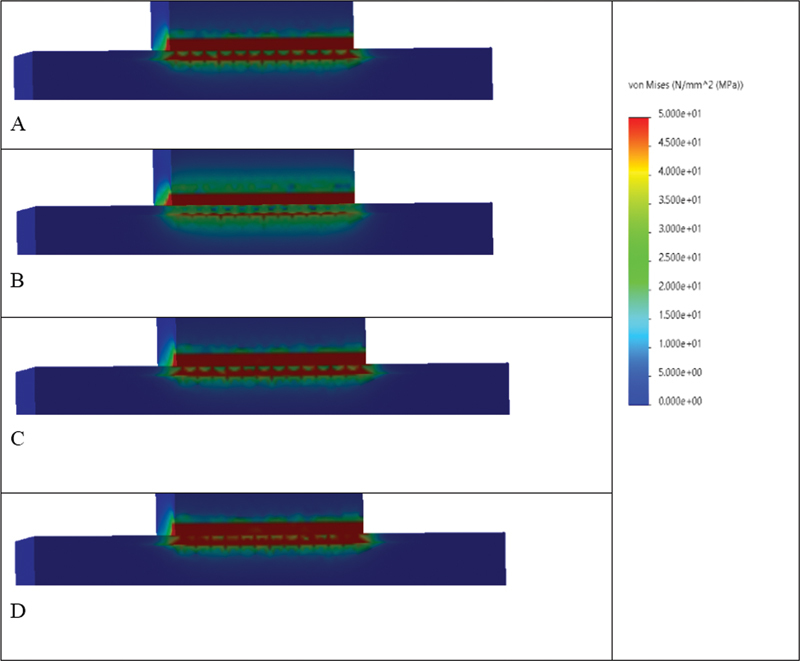
The von Mises stress distributions (MPa) at the interface region in model 1 (2 mm), presenting different material combinations ( **A** ) composite-to-composite, ( **B** ) ceramic-to-ceramic, ( **C** ) composite-to-tooth, ( **D** ) RMGI-to-tooth. Areas in red color present higher von Mises stress values, followed by areas in orange, yellow, and green. Areas in blue present the lowest von Mises stress values.

**Fig. 9:**
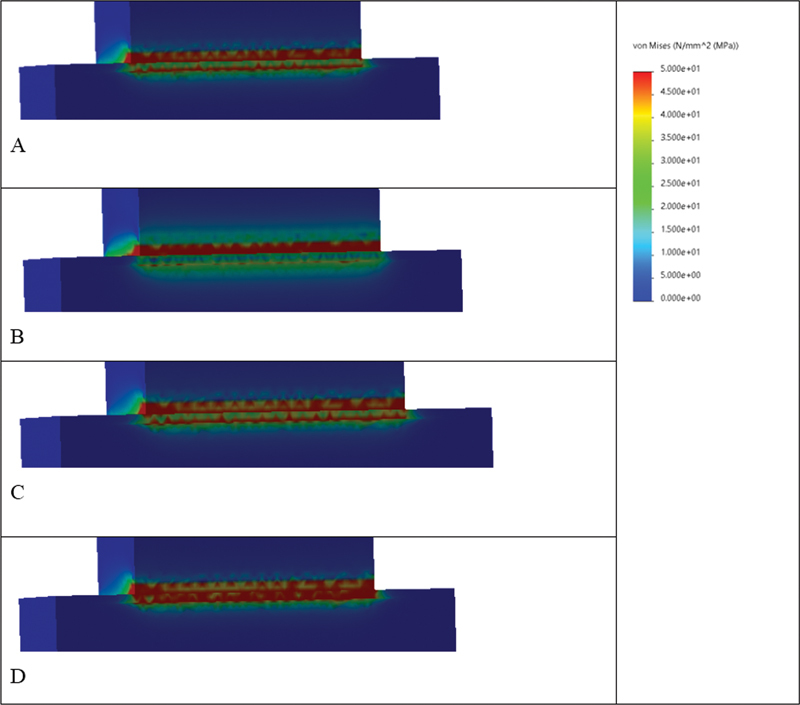
The von Mises stress distributions (MPa) at the interface region in model 1 (3 mm), presenting different material combinations. ( **A** ) Composite-to-composite, ( **B** ) ceramic-to-ceramic, ( **C** ) composite-to-tooth, ( **D** ) RMGI-to-tooth. Areas in red color present higher von Mises stress values, followed by areas in orange, yellow, and green. Areas in blue present the lowest von Mises stress values. It is evident that the highest von Mises stress values (red zones) are reported as follows: model D > C > A > B.

**Fig. 10:**
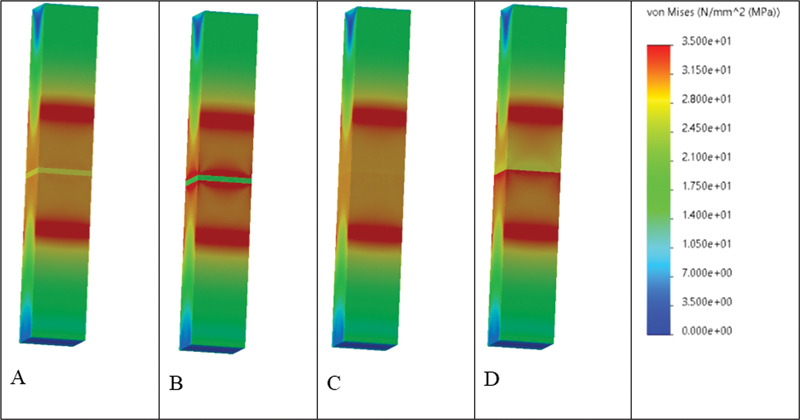
The von Mises stress distributions (MPa) in model 2 present different material combinations. ( **A** ) Composite-to-composite, ( **B** ) ceramic-to-ceramic, ( **C** ) composite-to-tooth, ( **D** ) RMGI-to-tooth. Areas in red color present higher von Mises stress values, followed by areas in orange, yellow, and green. Areas in blue present the lowest von Mises stress values. It is evident that von Mises stress is concentrated (red and orange zones) in the areas between the material interfaces and mid substrate body. The highest stress at the adhesive interfaces was reported in model D followed by model B.

Evaluation of the FEA results showed that von Mises stress was concentrated primarily at the cement layer and interfaces in Models 1 (2 mm and 3 mm) that simulate Slab_SBS, while it was distributed throughout the specimen in Model 2 that simulates μTBS, with the highest concentrations observed between the cement layer and mid substrate body ( Figs. 8 – 10 ). Model 1 (2 mm) showed an overall higher von Mises stress values compared to model 1 (3 mm); however, both showed similar stress distribution patterns for the different tested materials. Also, it worth noting that for models 1, RMGI-to-tooth models reported the highest stress concentration at the adhesive interfaces, followed by composite-to-tooth followed by composite-to-composite, whereas, the lowest stress concentration was observed in ceramic-to-ceramic models ( Figs. 8 and 9 and Table 4 ). Moreover, this was also noted in models simulating μTBS (models 2), representing RMGI-to-tooth that reported the highest von Mises stress values at the adhesive interface compared to other models. Nevertheless, other models reported higher stress concentration at the mid substrate body ( Fig. 10 and Table 4 ).

**Table 4:** The maximum von Mises stress values (N/mm ^2^ (MPa) for tested models and cement layers presenting different material combinations

Model/material	composite-to-composite		Ceramic-to-ceramic		Composite-to-tooth	RMGI-to-tooth
	Model	Adhesive layer	Model	Adhesive layer	Model	Model
Model 1 [2 mm]	86.3	86.3	75.2	39	87.8	98.2
Model 1 [3 mm]	73.1	64.7	69	36.2	74.4	81.5
Model 2 [1 mm]	44.8	29.6	45.1	25.9	44.7	44.6

## Discussion

Despite significant advancements in bond strength analysis, no ideal *in vitro* bond strength testing method is certified, which makes searching for new bond strength testing procedures necessary. 28 While it is true that the μTBS has been recognized as a reliable test to evaluate bond strength regardless of the tested material, the various modifications proposed by various researchers to the original microtensile methodology have resulted in inconsistent bond strength results for similar adhesive systems. 11 29 30

Multiple factors such as specimen shape and geometry, flaws during specimen preparation, the angle of loading, and variations in the tested materials' modulus of elasticity can have an impact on its results. 49 50 Furthermore, it is noteworthy that μTBS involves a significant number of methodological variables as it necessitates specialized testing jigs, a unique setup, rigorous testing procedures, and considered as a labor-intensive procedure. 51 52 In contrast, it is crucial to note that specimens' preparation for the innovative Slab_SBS in the current study is less labor-intensive because the samples are sectioned only in one direction into slabs rather than beams, and it is easier regarding handling and attachment.

It is important to point out that at the beginning of the current experiment, Slab_SBS resin composite to resin composite specimens of (1 mm) width were prepared and evaluated. However, it recorded no significant difference with the bond strength values of the Slab_SBS (2 mm and 3mm) with the same mode of failure ( Supplementary Table S1 and Fig. S2 , available in the online version). Therefore, for more ease of preparation and manipulation of specimens, Slab_SBS (1 mm) was not selected to be involved in the current study.

Although evaluation of the bond strength in the present study revealed that μTBS results are comparable to those obtained in the Slab_SBS (2 mm and 3mm), the μTBS triggers crack propagation into the substrate; therefore, a high percentage of cohesive and mixed failures were recorded 28 as contrast to the Slab_SBS that recorded merely adhesive failure. The cohesive failures resulting during microtensile test are considered valid if they are within 1 mm from the adhesive interface. 11 They should be included within the results analysis, which can alter the adhesive strength evaluation and are considered a drawback of microtensile bond strength technique. This result could be attributed to the fact that the bonded interface should uniformly be the stress-receiving zone, irrespective of the type of utilized bond strength test. 52 The high rate of cohesive and mixed failures observed is probably the result of passing of the loading force in the case of the μTBS protocol through the tooth substrate and the restorative material before reaching the adhesive interface, causing subsequent stress concentration at these sites. 31 On the contrary, in the Slab_SBS, loading forces are applied as close as possible to the interface, 52 leading to high stress concentrations near the targeted test site. 53

Based on the fact that *in vitro* tests provide the assessment of only one variable at a time while the other variables remain constant, and because the oral cavity is a dynamic environment, researchers cannot get accurate results if one or more variables are ignored. As a result, applications of FEA have increased substantially in the last decade as a method to evaluate mechanical characteristics, such as bond strength of dental materials, by imitating oral conditions. 54 55

The stresses generated by applied loads on a structure are calculated quantitatively using FEA. Stress on the tooth-restoration interface can be evaluated, and compressive forces are vertical to the restoration-tooth interface while tensile stresses have oblique direction 56 .

These results were confirmed by the FEA that predicts the stress distribution in the tested specimens based on their properties and how the load is applied. The stress analysis displayed that both bond strength-testing methods exhibited different stress distributions. In accordance with failure mode results, the Slab_SBS models showed uniform shear stress distribution in the bonded area; therefore, all its failures were adhesive. 57 Meanwhile, cohesive and mixed failures of the μTBS specimen are attributed to concentrations of high level of tensile stresses outside the bonding area. 58 This result is consistent with that of previous studies, in which researchers revealed that changing the direction of the applied force and the application area affects the distribution of the maximum von Mises stresses in the finite element stress analysis 59 60 . Therefore, the first null hypothesis of the current study was accepted, and the second one was rejected.

Consequently, it can be inferred that this result might have added a complimentary benefit of more consistency and reliability of the Slab_SBS. Based on these results, the Slab_SBS could be utilized as a reliable option for evaluating the bond strength of different substrates as it can provide comparable bond strength values and uniformly distribute the stresses.

Although the bond strength of the restorative materials can be evaluated in-vitro with the Slab_SBST, it has some limitations. For example, it involves application of unidirectional forces that do not exactly reflect the exact clinical situation, inconvenient application of specimens smaller than 2 mm width. Further investigation is required after different aging conditions to assess the validity of Slab_SBS after simulated aging.

## Conclusions

Under the limitation of the current study, it could be concluded that although μTBS was the standard in the past 20 years, the proposed Slab_SBS is easier to prepare and test with consistent, predictable outcome with no pretest failures during specimens' preparation and better stress distribution. Slab_SBS can be the future alternative to the conventional μTBS for evaluation of bond strength to save both time and effort.
