# Supplementary material for: Reliability of an Innovative Slab Shear versus Microtensile Bond Strength Test: Mechanical and Finite Element Analysis
Source: Eur J Dent. 2023 Apr 14;18(1):182–95. doi: 10.1055/s-0043-1763498 (PMC10959604; doi:10.1055/s-0043-1763498)
Supplement: Supplementary file 1 — Supplementary Material [file 10-1055-s-0043-1763498-s22112470.pdf]

Supplementary File

Materials and Methods

Slab shear bond strength [Slab_SBS] sample preparation is presented in Supplementary Fig. S1.

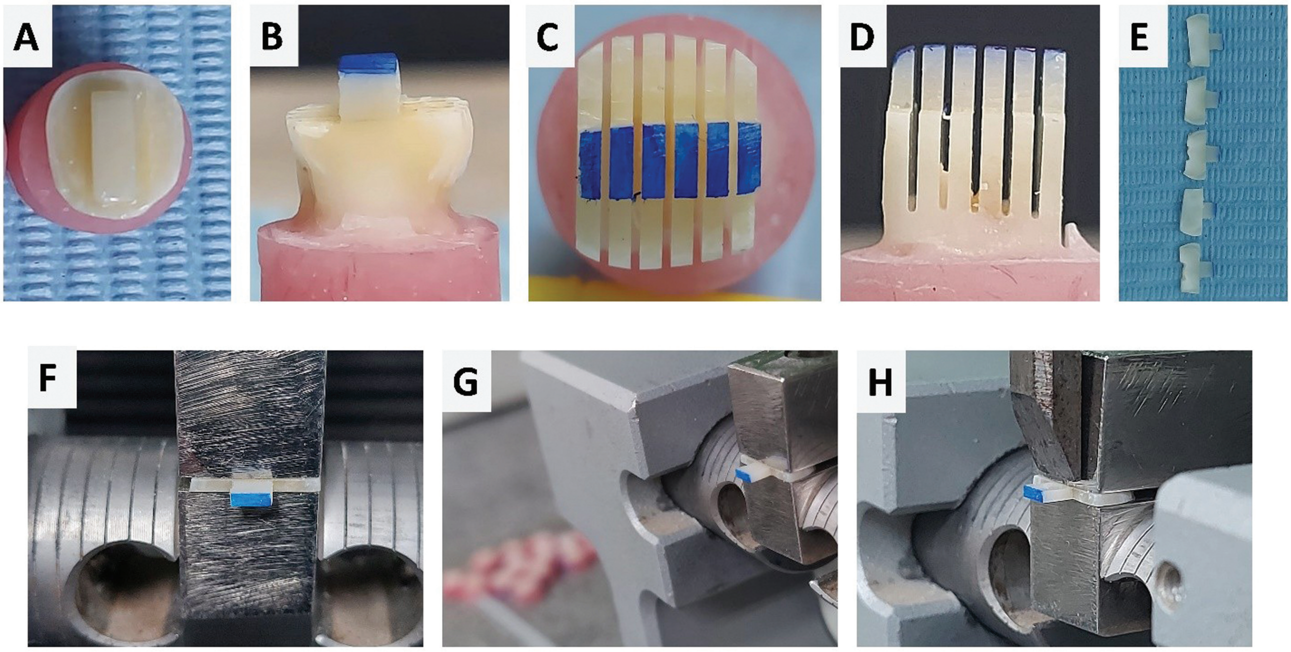

Supplementary Fig. S1 Steps for the preparation [A-E] and mounting [F-H] of the Slab_SBS samples. **A**: resin composite was built with 2mm thickness. B: The resin composite length was 3 mm, **C-D**: cutting the specimen into 1 mm thickness slabs using low speed diamond saw. **E**: Slab_SBS specimen after cutting and separation from the remining tooth structure. **F-G**: Mounting the Slab_SBS samples on the universal testing machine and load application [**H**].

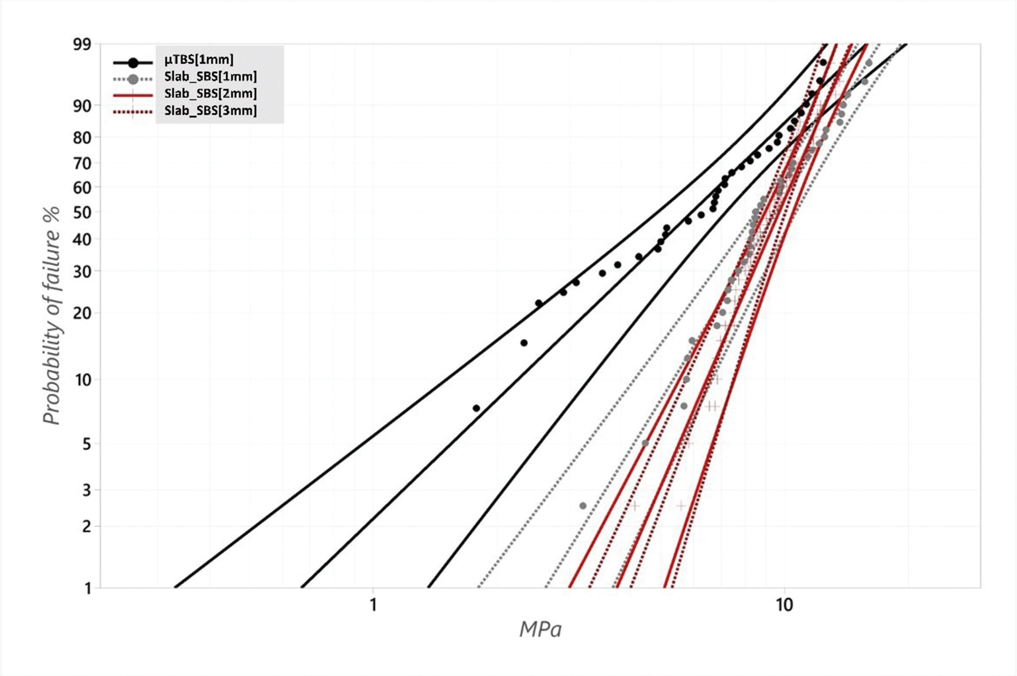

Supplementary Fig. S2 Weibull survival plot for composite-to-composite substrate.

 © 2023. The Author(s).

Results of composite-to-composite substrate for Slab_SBS [1 mm] group

The results of Slab_SBS [1 mm] was coherent with Slab_SBS [2 mm] and Slab_SBS [3 mm]. Although Slab_SBS [1 mm] resulted in better values compared to µTBS[1mm], sample preparation with that size was difficult.

Supplementary Table S1 Results of Weibull analysis for composite-to-composite substrate

Substrate	Method/size	pft	α [95% CI]	β [95% CI]	P10 [95% CI]	FA[A/C/M]
Composite-to-composite	µTBS [1 mm]	6/40	7.2[6.1 to 8.5][a]	1.9[1.5 to 2.5][a]	2.1[1.5 to 3.3][a]	[80/5/15]
Composite-to-composite	Modi-slab_SBS [1 mm]	0/40	10.7[9.7 to 11.8][b]	3.3[2.6 to 4.2][b]	5.4[4.3 to 6.7][b]	[100/0/0]
Composite-to-composite	Modi-slab_SBS [2 mm]	0/40	10.5[9.8 to 11.3][b]	4.7[3.7 to 5.9][b]	6.5[5.6 to 7.5][b]	[100/0/0]
Composite-to-composite	Modi-slab_SBS [3 mm]	0/40	10.1[9.5 to 10.7][b]	5.3[4.2 to 6.7][b]	6.6[5.8 to 7.5][b]	[100/0/0]

 © 2023. The Author(s).
